# Fertility preservation in prepubertal boys: management and follow-up challenges

**DOI:** 10.1530/EC-26-0146

**Published:** 2026-06-09

**Authors:** Vittorio Ferrari, Federico Baronio, Marcello Lanari, Rod T Mitchell

**Affiliations:** ^1^ Pediatric Unit, IRCCS Azienda Ospedaliero-Universitaria di Bologna, Bologna, Italy; ^2^ Department of Medical and Surgical Sciences, Alma Mater Studiorum, University of Bologna, Bologna, Italy; ^3^ Royal Hospital for Children and Young People, Edinburgh, UK; ^4^ Centre for Reproductive Health, Institute of Regeneration and Repair, University of Edinburgh, Edinburgh, UK

**Keywords:** fertility preservation, prepubertal boys, testicular tissue cryopreservation, gonadotoxic therapy, spermatogonial stem cells

## Abstract

Survival after childhood cancer and severe haematological disorders has improved substantially, shifting clinical attention towards long-term quality-of-life outcomes. Preservation of male reproductive potential represents a major challenge, particularly in boys exposed to gonadotoxic therapies before the onset of spermatogenesis. While sperm cryopreservation is an established fertility preservation strategy for postpubertal males, no clinically validated option currently exists for prepubertal boys. Male fertility depends on the establishment and lifelong maintenance of a finite spermatogonial stem cell (SSC) pool within a specialized somatic microenvironment. Although the prepubertal testis lacks active spermatogenesis, it already contains the SSC populations required for future fertility and these are vulnerable to cytotoxic injury. Clinical and histological evidence shows that chemotherapy and radiotherapy, including conditioning regimens for haematopoietic stem cell transplantation, can deplete SSCs even when exposure occurs in early childhood, with consequences that may become apparent only at puberty or later in adulthood. Testicular tissue cryopreservation (TTC) has emerged as the only potential fertility preservation strategy for prepubertal boys at high risk of treatment-induced infertility. TTC aims to preserve immature testicular tissue containing SSCs for possible future use through experimental approaches. However, no human pregnancies have yet been achieved using tissue cryopreserved before puberty, and all current applications remain experimental. This review summarizes current practice for TTC in prepubertal boys, with a particular focus on the issues most relevant to paediatric endocrinologists involved in the long-term follow-up and management of these patients.

## Introduction

Survival rates after childhood cancer and severe haematological disorders have improved over recent decades, with 5-year survival rates above 80% (1). As increasing numbers of boys reach adulthood, long-term quality-of-life outcomes have become a central component of survivorship care. Among these, impairment of reproductive potential represents one of the most frequent and distressing late effects reported by male survivors (2).

In postpubertal males, sperm cryopreservation is a well-established strategy that should be systematically offered prior to gonadotoxic therapy (3). The major unresolved clinical issue concerns boys treated before puberty, whose testes do not yet produce mature spermatozoa and therefore cannot benefit from conventional sperm banking. In these patients, fertility preservation is limited to experimental approaches aimed at preserving the spermatogonial stem cell (SSC) pool, the cellular basis of lifelong spermatogenesis.

Clinical decision-making in this setting is complex and is further complicated by uncertainty regarding individual infertility risk and treatment-related gonadotoxicity. Permanent fertility impairment is primarily determined by quantitative or functional loss of SSCs; however, future reproductive capacity also depends on preservation of the somatic testicular microenvironment that supports germ cell survival, differentiation, and maturation (4).

Contrary to earlier assumptions, the prepubertal testis is not resistant to gonadotoxic injury. Clinical and histological studies have shown significant reductions in spermatogonial number and tubular fertility index in boys exposed to alkylating agents or radiotherapy, even when treatment occurs before puberty (5, 6). These observations indicate that damage to the SSC pool during childhood may result in irreversible impairment of future fertility.

In this context, testicular tissue cryopreservation (TTC) has been proposed as a fertility preservation option for selected prepubertal boys at high risk of treatment-induced infertility. TTC aims to preserve immature testicular tissue containing SSCs for possible future use, although no clinically validated method for restoring fertility from tissue cryopreserved before puberty is currently available (3, 7).

This review provides an overview of current evidence and clinical practice regarding TTC in prepubertal boys and examines the key clinical issues from the perspective of a paediatric endocrinologist, with a focus on gonadotoxic testicular damage, its implications for clinical decision-making and counselling, and long-term pubertal and gonadal follow-up.

### Search strategy and selection criteria

References for this review were identified through searches of PubMed, with an emphasis on studies published over the past 25 years. Searches used combinations of terms related to TTC, fertility preservation, prepubertal boys, SSCs, and gonadotoxic therapies. Additional relevant articles were identified through reference lists of key publications and documents from international scientific societies and consensus groups. Only English-language, peer-reviewed articles were considered. Clinical, histological, experimental, and translational studies, as well as guidelines and expert consensus papers, were included. Abstract-only publications, non-peer-reviewed articles, and sponsored supplements were excluded. References were selected based on relevance, scientific quality, and contribution to the understanding of fertility preservation in prepubertal males.

### Biological basis of fertility vulnerability in boys exposed to gonadotoxic therapies

Male fertility relies on the establishment and lifelong maintenance of a functional SSC pool within the seminiferous epithelium. During fetal development, primordial germ cells differentiate into gonocytes within the developing seminiferous cords under the influence of a regulated somatic microenvironment composed of Sertoli cells, peritubular myoid cells, and Leydig cells (8). A transient activation of the hypothalamic-pituitary-gonadal (HPG) axis during early postnatal life (‘mini-puberty’) promotes gonocyte migration towards the basement membrane and differentiation into spermatogonia, including establishment of the SSC pool that will sustain spermatogenesis throughout adulthood (9). From infancy until puberty, SSCs persist in a relatively quiescent state, undergoing limited self-renewal, while completion of spermatogenesis requires reactivation of the HPG axis at puberty (10). Although SSCs undergo self-enewal, their long-term maintenance and developmental competence depend on regulated interactions with the somatic compartment of the seminiferous epithelium. Sertoli cells and peritubular myoid cells provide structural support, while Leydig cells contribute paracrine hormone signalling required to maintain SSC number and function (11).

**Figure 1:**
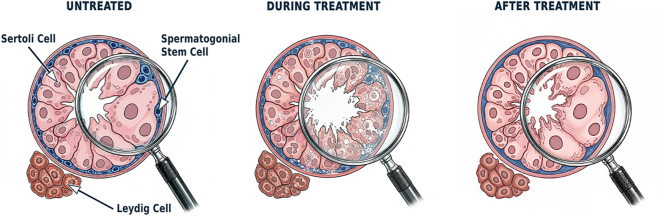
Schematic representation of gonadotoxic injury in the prepubertal testis. The figure illustrates a seminiferous tubule in the untreated state, during gonadotoxic exposure, and after treatment. In the untreated prepubertal testis, SSCs are distributed along the basement membrane and represent the cellular reservoir for future spermatogenesis. During gonadotoxic exposure, proliferating germ cells are preferentially damaged, with associated injury to the seminiferous epithelium and possible involvement of the surrounding somatic and interstitial compartments. After treatment, depletion of the SSC pool together with residual architectural alterations of the seminiferous tubule and interstitium may contribute to long-term impairment of spermatogenic potential; when SSCs survive, their capacity to reinitiate spermatogenesis may also be impaired.

The low proliferative activity of the prepubertal testis originally led to the assumption that it might be relatively protected from cytotoxic injury. However, this view has been consistently challenged by clinical and histological evidence. Testicular biopsies from boys exposed to gonadotoxic therapies before puberty demonstrate significant reductions in spermatogonial number and tubular fertility index compared with age-matched controls (5, 6). These findings indicate that the prepubertal testis is already vulnerable during childhood and that damage incurred at this stage may irreversibly compromise future reproductive potential. Because the prepubertal testis contains the only germ cell reservoir available for future spermatogenesis, quantitative depletion or qualitative impairment of SSCs may prevent adequate spermatogenic recovery when the HPG axis is reactivated at puberty (Fig. 1) (4). Differentiating spermatogonia are highly susceptible to chemotherapy and radiotherapy because of their mitotic activity and limited DNA repair capacity. Although SSCs are relatively more resistant, owing to their ability to arrest the cell cycle and activate DNA repair pathways, they are not protected from injury (12). Long-term infertility may therefore result not only from quantitative depletion of the SSC pool but also from qualitative impairment of surviving SSCs, including reduced clonogenic capacity or persistence of DNA damage induced by cytotoxic therapies (4, 13).

In addition to direct germ cell loss, gonadotoxic therapies may also impair the somatic cell populations that regulate the SSC niche and support later spermatogenesis (14). Accordingly, even when some SSCs survive, impaired somatic support may limit their capacity to sustain later spermatogenic recovery, thereby amplifying the long-term effects of gonadotoxic therapies (15). The prepubertal testis, therefore, contains all the cellular prerequisites for future spermatogenesis but lacks any meaningful capacity for regeneration once the SSC pool is compromised. Injury sustained during childhood may therefore remain clinically silent for years and become apparent only with pubertal maturation, when impaired spermatogenesis or other manifestations of gonadal dysfunction may first emerge (16).

### Treatments and conditions associated with high risk of permanent infertility

The risk of long-term or permanent gonadal dysfunction and infertility following childhood cancer or severe haematological disease is variable and depends on both treatment-related exposures and disease-related factors (17, 18).

#### Chemotherapy-associated risk

Alkylating agents represent the chemotherapeutic class most strongly associated with permanent infertility. Cyclophosphamide, busulfan, melphalan, ifosfamide, and procarbazine induce DNA crosslinks that interfere with replication and transcription, leading to apoptosis in rapidly dividing cells. Because spermatogonia, including SSCs, are particularly sensitive to DNA damage, cumulative exposure to alkylating agents is closely associated with germ cell depletion (2, 19, 20). To facilitate risk stratification, cyclophosphamide equivalent dose (CED) has been developed as a standardized metric that integrates exposure to different alkylating agents into a single cumulative dose estimate based on the relative toxicity of each alkylating agent. Clinical studies have demonstrated that higher cumulative exposure to alkylating agents is associated with an increased risk of azoospermia, particularly at CED values above 8 g/m^2^; however, risk increases progressively across exposure ranges, and substantial interindividual variability remains. CED should therefore be interpreted in the context of combined treatment exposures rather than as a stand-alone predictor of outcome (3, 13).

Platinum-based agents, including cisplatin and carboplatin, induce DNA crosslinks and have been shown to impair spermatogenesis in experimental models and clinical studies, although their gonadotoxicity is not yet fully captured by current risk prediction models, potentially leading to underestimation of fertility risk in treated patients (21, 22).

Other classes of chemotherapeutic agents, including antimetabolites, anthracyclines, and microtubule-targeting drugs, are generally associated with a lower risk of permanent infertility when used as single agents but may contribute to clinically relevant gonadotoxicity within multi-agent protocols (17, 18).

In addition, the long-term reproductive impact of newer targeted therapies and immunotherapies remains incompletely defined. Although these agents are increasingly incorporated into paediatric treatment protocols, available human data on their gonadotoxic potential are limited, making individualized risk assessment and counselling particularly difficult in this setting (23).

#### Radiotherapy and conditioning regimens

The testis is among the most radiosensitive organs. Temporary azoospermia has been reported after exposures of 2–3 Gy, while permanent infertility is commonly observed at doses exceeding 6 Gy (4). Even low-dose scatter radiation during pelvic or abdominal radiotherapy can contribute to germ cell loss (24).

Total body irradiation (TBI), frequently used as part of conditioning regimens for haematopoietic stem cell transplantation (HSCT), carries a particularly high risk of permanent infertility. Typical regimens use fractionated TBI schedules around 9.9–13.2 Gy, and long-term follow-up reports azoospermia in up to ∼85% of treated males (25). Although reduced-intensity conditioning has been introduced to mitigate late effects, available evidence indicates that gonadotoxic risk remains substantial (26).

Cranial irradiation may impair later reproductive function through a central mechanism, by damaging the hypothalamic-pituitary axis rather than the testes directly. Injury to hypothalamic GnRH neurons or pituitary gonadotrophs may result in hypogonadotropic hypogonadism despite structurally intact testes (27). This dysfunction may become apparent only years later, particularly in boys treated before puberty, in whom physiological quiescence of the HPG axis may delay recognition until the expected time of pubertal onset (16).

#### Disease-related factors

Increasing evidence indicates that the underlying disease may compromise germ cell number even before therapy begins. Reduced spermatogonial counts at diagnosis have been reported in boys with central nervous system tumours, haematological malignancies, and non-malignant conditions, such as sickle cell disease and thalassaemia (6, 28). Although the mechanisms underlying this pre-treatment depletion remain incompletely defined, baseline germ cell impairment may amplify the gonadotoxic effects of subsequent therapy and complicate individualized risk assessment, counselling, and longitudinal interpretation of gonadal outcomes (29).

### Fertility preservation across pubertal stages

Fertility preservation strategies in boys and young men exposed to gonadotoxic therapies are determined primarily by pubertal status. The transition into puberty is clinically relevant because it determines whether sperm-based fertility preservation options are available or whether experimental tissue-based strategies must be considered (3). Accordingly, fertility preservation in postpubertal patients relies on standard-of-care interventions, whereas options for prepubertal boys are limited to experimental strategies.

In pubertal and postpubertal males, sperm cryopreservation is the gold standard and should be systematically offered before gonadotoxic therapy (3). Testicular volume is particularly informative in this setting, as it correlates with successful semen collection: motile spermatozoa have been identified in ejaculates at volumes as low as 6–7 mL, whereas in a biopsy series, mature intratesticular sperm were found only in boys with testicular volumes >10 mL (30, 31). For this reason, accurate clinical assessment of pubertal stage is essential, particularly in boys approaching or entering puberty, in whom even limited sperm production may open access to established fertility preservation options. Cryopreserved sperm can be stored long term and later used for assisted reproductive techniques, including intracytoplasmic sperm injection (ICSI), enabling successful fertilization even with very limited numbers of spermatozoa (3, 32).

When semen collection by masturbation is not feasible, alternative approaches, such as penile vibratory stimulation, electroejaculation, or surgical sperm retrieval techniques, including testicular sperm extraction (TESE), may be used. Although more invasive, these methods can yield motile sperm and are recommended when conventional semen collection is unsuccessful (3).

Fertility preservation is optimally performed before treatment initiation; attempts after exposure to gonadotoxic agents are less reliable because spermatogenesis may already be impaired (33). Early referral and timely counselling are therefore critical.

In contrast, fertility preservation in prepubertal boys poses a distinct challenge because the testes do not yet produce mature spermatozoa, precluding conventional sperm banking, and decisions must therefore be made before future reproductive function can be clinically assessed (3). Despite the presence of SSCs, no clinically applicable fertility preservation strategy based on these cells is currently available. TTC has been proposed as an experimental strategy for boys at high risk of treatment-induced infertility (34). Over the past two decades, specialized programmes in Europe and North America have offered TTC within structured research protocols, reflecting both the anticipated high risk of infertility in selected patients and the absence of validated alternatives. However, all current methods intended to generate viable gametes from cryopreserved testicular tissue remain experimental. To date, no human pregnancies have been achieved using tissue cryopreserved before puberty. A first-in-human preprint report has described graft survival and histological evidence of spermatogenesis after autologous transplantation of cryopreserved immature testicular tissue in a single patient (35). However, this observation remains preliminary, and no method for restoring spermatogenesis from prepubertal tissue has been validated for clinical use (3, 7). TTC should therefore still be considered an experimental strategy rather than a clinically established fertility preservation intervention.

### Testicular tissue cryopreservation in prepubertal boys: current practice and limitations

**Figure 2:**
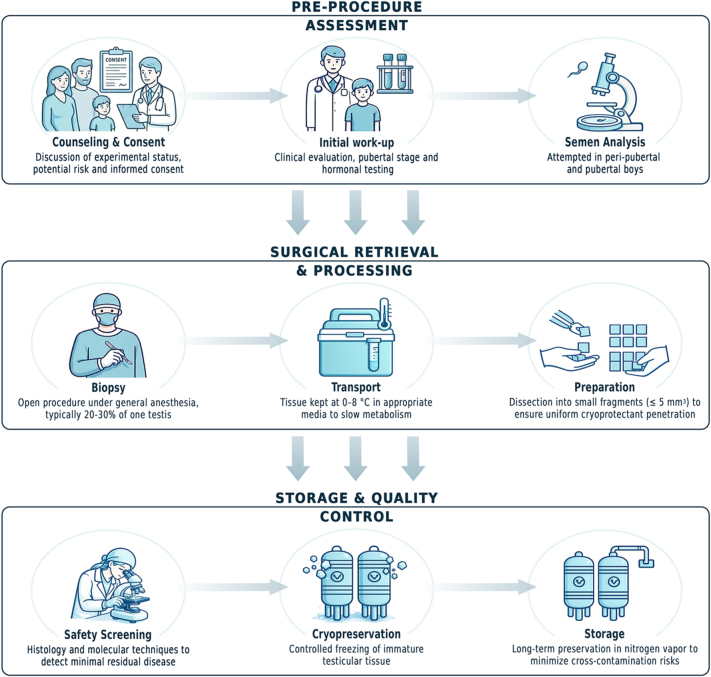
Overview of the clinical workflow for TTC in prepubertal boys. The figure outlines the sequence of steps leading from pre-procedure assessment to tissue retrieval, processing, and long-term cryostorage. Before biopsy, the pathway includes counselling and informed consent, along with baseline evaluation of pubertal stage and other relevant clinical and laboratory parameters; semen analysis is added when appropriate in peri-pubertal or pubertal boys. Testicular tissue is then obtained under general anaesthesia, often during another clinically indicated procedure, such as central venous catheter placement, transported under controlled conditions, and processed into fragments for cryopreservation. The final phase comprises safety checks, freezing, and long-term storage. TTC is currently offered as an experimental fertility preservation option within approved clinical research programmes.

TTC is currently the only experimental fertility preservation strategy available for prepubertal boys at high risk of treatment-induced infertility and should be offered exclusively within ethically approved research protocols and performed in specialized centres, with integrated oncologic, haematologic, endocrine, and reproductive expertise. Standard practice therefore requires careful patient selection, defined surgical and laboratory procedures, and long-term follow-up (Fig. 2) (3, 36). However, several practical aspects of TTC, including eligibility thresholds, tissue handling, quality control, and follow-up, remain incompletely standardized and continue to rely partly on expert consensus and centre-specific protocols (3).

#### Patient selection and eligibility

Appropriate patient selection is essential to ensure that TTC is offered to boys with a significant risk of treatment-induced infertility who cannot benefit from conventional sperm banking (Table 1). Risk stratification is largely based on the expected gonadotoxic burden of planned therapy, particularly cumulative exposure to alkylating agents and conditioning regimens for HSCT (Table 2). According to current recommendations, boys exposed to high-risk regimens, particularly those involving high cumulative alkylating exposure, TBI, or direct testicular irradiation, represent the group most likely to benefit from TTC and should be systematically counselled about this option (3).

**Table 1:** Overview of patient selection for TTC in boys.

Category	When to consider TTC	When not appropriate
Age and development	Prepubertal or early pubertal boys (Tanner stages I–II) unable to provide a semen sample	Established sperm-based options available
Treatment risk	Planned treatment with high gonadotoxic risk (e.g. CED > 8 g/m^2^, TBI, or testicular radiation)	Planned treatments with low gonadotoxic risk
Medical condition	Malignant diseases (leukaemias, lymphomas, and sarcomas) or non-malignant conditions requiring HSCT	Very poor short-term survival prognosis
Surgical fitness	Patient stable enough to undergo general anaesthesia and a minor surgical procedure	Prohibitive surgical/anaesthetic risk
Consent	Informed consent provided by parents/legal guardians (and patient assent where applicable)	No parental consent/refusal of experimental procedure

CED, cyclophosphamide equivalent dose; HSCT, haematopoietic stem cell transplantation; TBI, total body irradiation; TTC, testicular tissue cryopreservation.

**Table 2:** Risk stratification for TTC in boys. This table provides a schematic overview of risk stratification for clinical orientation; however, individual risk remains influenced by treatment combinations, underlying disease, and the limited evidence supporting fixed eligibility thresholds (3).

Risk category	Therapeutic exposure	TTC recommendation
High risk	CED > 8 g/m^2^, TBI, or direct testicular radiation (>6 Gy)	Should be offered within fertility preservation research protocols
Intermediate risk	4 ≤ CED ≤ 8 g/m^2^	May be considered on an individual basis
Low risk	CED < 4 g/m^2^	Not routinely indicated in the absence of additional risk factors

CED, cyclophosphamide equivalent dose; TBI, total body irradiation; TTC, testicular tissue cryopreservation.

Beyond oncology, TTC is increasingly considered in boys with non-malignant conditions requiring gonadotoxic therapies, such as sickle cell disease, thalassaemia major, and severe immunodeficiencies (37). These patients often have excellent long-term survival, making fertility preservation particularly relevant despite the experimental nature of TTC.

While no formal contraindications to testicular biopsy have been established, proposed exclusion criteria include patients receiving exclusively palliative care, those who are medically unfit for surgery or anaesthesia, or situations in which bleeding or infection risk render biopsy unsafe. TTC should also not replace conventional sperm cryopreservation in peri- or postpubertal patients when viable spermatozoa can be obtained (3).

#### Pre-procedure assessment

Pre-procedure assessment aims to confirm eligibility and optimize timing of TTC. Evaluation includes clinical history and assessment of pubertal stage, including measurement of testicular volume to document baseline testicular status before testicular biopsy and gonadotoxic treatment in order to support interpretation of post-treatment growth and possible treatment- or biopsy-related damage, identify any pre-existing abnormalities, and establish whether conventional sperm-based fertility preservation may already be feasible (30, 31). Although pre-procedure hormonal assessment has a limited stand-alone interpretative value in prepubertal boys, because gonadotrophins and testosterone are physiologically low during childhood, it may still be informative as part of baseline characterization for subsequent post-treatment follow-up. In this context, baseline inhibin B, for which age-related normative data are available, may provide additional information on Sertoli cell function and serve as an indicator of pre-existing testicular dysfunction as well as a reference point against which treatment- or biopsy-related changes can later be assessed (38). In late childhood, endocrine findings may also complement clinical assessment by providing further insight into developmental status and by helping to determine whether established sperm-based fertility preservation options should also be considered. Accordingly, in peri- or postpubertal patients, semen analysis should always be considered before considering TTC, with extended analysis following centrifugation when initial samples are azoospermic, in accordance with WHO recommendations (39). Overall, pre-procedure assessment remains primarily based on clinical evaluation, and the contribution of endocrine testing before puberty is limited and context-dependent (3); however, in the experimental setting of TTC and given that the gonadal impact of specific cancer treatment regimens cannot always be fully anticipated at the individual level, a broader baseline assessment may still be valuable for later follow-up.

#### Surgical retrieval of testicular tissue

Surgical retrieval of immature testicular tissue is performed as a standardized, minimally invasive procedure aimed at maximizing tissue yield while minimizing complications (3). A unilateral open testicular biopsy under general anaesthesia is recommended, preferably combined with another clinically indicated procedure (such as central venous line placement or bone marrow aspiration) to avoid additional anaesthetic exposure (36).

Sampling of a single testis is usually sufficient to obtain adequate tissue for cryopreservation while preserving contralateral testicular integrity. Removal of 20–30% of one testis provides sufficient seminiferous tubules for processing and storage without impairing subsequent testicular growth or endocrine function (3). Bilateral biopsies are generally avoided and should be considered only in exceptional circumstances.

The biopsy is usually taken from the equatorial region of the testis, where seminiferous tubules are abundant and vascular injury can be minimized. Immediately after excision, tissue is placed in appropriate transport medium and transferred promptly to the laboratory for processing (3).

In peri- or postpubertal patients unable to provide an ejaculate, surgical sperm retrieval techniques, such as TESE or micro-TESE, may be attempted first, with cryopreservation of testicular tissue performed during the same procedure if no spermatozoa are identified (36).

Reported complication rates associated with testicular tissue biopsy are low. In a large international survey, the overall complication rate was approximately 7%, with wound infection occurring in less than 1% of cases and clinically significant bleeding reported in only 0·1% (36). When performed according to current recommendations, no long-term adverse effects on testicular growth or pubertal development have been documented (3, 36).

#### Handling and cryopreservation of testicular tissue

Following surgical retrieval, the testicular tissue must be transported and processed under controlled conditions within defined timeframes to preserve spermatogonial viability and seminiferous architecture. Most protocols recommend tissue processing within 24 h of biopsy, with maintenance at 0–8°C to slow cellular metabolism and limit hypoxic and enzymatic damage while avoiding premature freezing injury prior to cryopreservation (7).

Upon arrival in the laboratory, testicular tissue is typically dissected into small fragments to allow uniform penetration of cryoprotective agents and consistent cooling during freezing. Most centres process tissue into fragments ≤5 mm^3^, a size that facilitates cryoprotectant diffusion and improves freezing kinetics (3). At this stage, a portion of the tissue is commonly allocated for histological and immunohistochemical assessment to confirm the presence and distribution of spermatogonia and to provide baseline information on tissue quality, while the remaining fragments are prepared for cryopreservation according to centre-specific protocols (7). However, approaches to histological processing, germ cell quantification, and marker selection remain heterogeneous across centres, which complicates the direct comparison of tissue quality between programmes (3, 36).

Slow freezing currently represents the most widely adopted and best characterized cryopreservation strategy for human immature testicular tissue, relying on controlled cooling rates and the use of cryoprotective agents (7). Controlled-rate freezing using programmable freezers is generally preferred to improve reproducibility, whereas uncontrolled slow freezing may be associated with greater variability in post-thaw tissue quality (3).

Vitrification, an ultra-rapid freezing technique that avoids ice crystal formation by transitioning tissue into a glass-like state, has shown promising results in animal models. However, experience with vitrification of human immature testicular tissue remains limited (7).

Following freezing, testicular tissue fragments are stored at ultra-low temperatures in liquid nitrogen, either in the liquid or in the vapour phase. Storage in nitrogen vapour is often favoured for safety reasons, as it reduces the risk of cross-contamination while ensuring long-term tissue stability (3).

Despite increasing experience, no single cryopreservation protocol has yet been universally adopted or validated as superior, and current practice remains partly shaped by local protocols and centre-specific expertise (3, 7).

#### Safety and quality control

Oncological safety represents the principal barrier to the clinical translation of TTC, particularly because of the risk of malignant contamination in tissue obtained from boys with cancer (3, 7). This concern is particularly relevant in haematological malignancies, where malignant cells may infiltrate the testes or persist as minimal residual disease. Malignant contamination has been documented in a substantial proportion of testicular tissue samples from boys with haematological malignancies, with reported rates of up to 37%, and lower but non-negligible rates described in selected solid tumours (3, 40, 41).

Several complementary approaches are used to assess oncological safety of cryopreserved tissue. Conventional histology allows detection of overt malignant infiltration but has limited sensitivity for low-level disease. Immunohistochemistry improves sensitivity by identifying tumour-associated markers and is routinely incorporated into quality control protocols in many centres. Molecular techniques, including PCR-based detection of clonal immunoglobulin or T cell receptor gene rearrangements, further increase sensitivity and are particularly relevant in haematological malignancies, where MRD may persist despite apparent remission (3, 40). In research settings, xenotransplantation of human testicular tissue into immunodeficient mice has been explored as a functional bioassay to detect occult malignant contamination (40).

Quality control also includes assessment of tissue integrity and germ cell content. Histological evaluation of seminiferous architecture and immunohistochemical staining for germ cell markers allow identification and quantification of spermatogonia (7). However, current methodologies remain incompletely standardized, particularly with respect to quantification methods and marker selection (3). Quantitative approaches based on age-adjusted reference values, including Z-scores for spermatogonial number, have been shown to accurately distinguish physiological age-related variability from pathological germ cell depletion (3). However, this approach relies on an optimal number of round tubular cross sections, which are often limited. Therefore, quantification methods that normalize germ cell counts to total tubular area, rather than relying on round tubular cross sections, have been validated in human fetal and prepubertal testicular tissue. These have been shown to correlate with spermatogonial counts per tubule when these can be reliably assessed, supporting their use in quality assessment of cryopreserved tissue (42).

Despite these measures, complete exclusion of malignant contamination cannot currently be guaranteed, especially in patients with haematological malignancies. This limitation represents a major barrier to clinical application of autologous tissue transplantation, which may potentially be overcome by further progress in *in vitro* spermatogenesis (3).

#### Follow-up and long-term monitoring

Follow-up after TTC focuses on longitudinal monitoring of pubertal development and gonadal function while maintaining clinical continuity for any potential future use of cryopreserved tissue (3). Because TTC is performed before the onset of spermatogenesis, follow-up during childhood is mainly observational. This period remains clinically relevant, as the first signs of altered pubertal development or gonadal dysfunction may become apparent only at the expected time of pubertal onset (16).

During childhood and adolescence, follow-up should include repeated clinical assessment of pubertal progression, testicular growth, and endocrine function. Clinical examination with Tanner staging and testicular volume measurement remains central, while biochemical monitoring of gonadotrophins and testosterone becomes informative only after pubertal activation; although HPG axis abnormalities are not detectable in early childhood, rising gonadotrophins during pubertal progression may indicate primary testicular dysfunction (43). Additional biomarkers, such as inhibin B and anti-Müllerian hormone (AMH), may provide complementary information on Sertoli cell function, although their predictive value for future fertility remains uncertain (44). In particular, recent prospective data suggest that inhibin B may be useful as a longitudinal marker of emerging Sertoli cell dysfunction after treatment, as levels were generally preserved before and during puberty, but more often fell below the reference range in the postpubertal period; by contrast, AMH remained largely stable over time and appears less informative for tracking evolving gonadal damage (45).

However, normal pubertal progression does not exclude significant impairment of spermatogenesis, highlighting the dissociation between pubertal development and future reproductive potential (46, 47).

In postpubertal survivors, semen analysis remains the gold standard for assessing spermatogenic status, including in individuals who underwent TTC in childhood (4). Normal or recovering spermatogenesis may obviate the need for experimental use of stored tissue.

At present, no standardized follow-up protocols exist for experimental fertility restoration procedures, as autologous transplantation of prepubertal testicular tissue has not yet resulted in restored fertility in humans (7). Participation in long-term registries is therefore essential to evaluate the long-term safety, feasibility, and potential benefit of TTC and to inform future clinical guidance and counselling (48). Long-term follow-up may also include counselling and obtaining renewed consent from the patient regarding the future management of cryopreserved tissue. This is particularly important when the initial consent was provided by the parents and the patient has reached an age at which they have the capacity to consent (3).

### Experimental approaches to fertility restoration and current limitations

**Figure 3:**
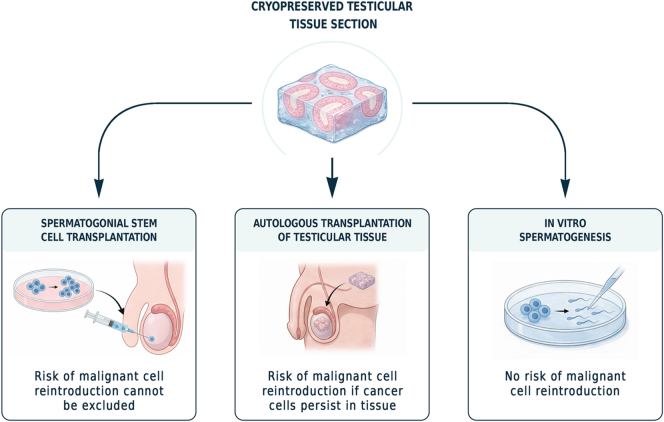
Experimental strategies for fertility restoration using cryopreserved immature testicular tissue. Schematic overview of the three main experimental approaches currently under investigation for fertility restoration from testicular tissue cryopreserved before puberty: SSC transplantation, autologous grafting of cryopreserved immature testicular tissue, and *in vitro* spermatogenesis. The figure highlights key conceptual differences between approaches, particularly regarding the risk of malignant cell reintroduction, which remains a major barrier to clinical translation.

Current experimental research explores three main approaches: transplantation of SSCs, autologous grafting of cryopreserved testicular tissue, and *in vitro* spermatogenesis (Fig. 3) (3, 7).

#### Spermatogonial stem cell transplantation

SSC transplantation aims to reintroduce a patient’s own SSCs into the seminiferous tubules, where they may engraft within the seminiferous epithelium and potentially support spermatogenesis. In animal models, this approach has successfully restored spermatogenesis and resulted in the birth of healthy offspring, demonstrating proof-of-principle for SSC-based fertility restoration (11, 49).

In humans, technical feasibility has been demonstrated in *ex vivo* studies showing that injection of cell suspensions into the rete testis can achieve widespread intratesticular distribution (50, 51). However, clinical translation faces major challenges. The number of SSCs available in small prepubertal biopsies is extremely limited, making *in vitro* expansion a prerequisite for transplantation. Although culture systems capable of expanding human SSCs have been reported, results have been inconsistent, and uncertainties remain not only regarding long-term genetic and epigenetic stability but also regarding the ability of expanded cells to re-establish functional spermatogenesis after transplantation (52, 53, 54).

Oncological safety represents an additional barrier. SSC suspensions derived from boys with haematological malignancies may contain malignant cells, and current purification strategies (including magnetic-activated and fluorescence-activated cell sorting) have not reliably eliminated this risk in human samples (55, 56, 57). Consequently, SSC transplantation raises significant concerns of neoplastic cell dissemination, particularly in patients treated for leukaemia or lymphoma, and remains confined to experimental research settings.

#### Autologous transplantation of testicular tissue fragments

Autologous transplantation of cryopreserved testicular tissue fragments preserves the native somatic microenvironment that supports germ cell maturation. In animal models, including non-human primates, grafted tissue has survived long term, resumed spermatogenesis, and resulted in live offspring, providing the strongest preclinical evidence for this approach (58, 59).

In humans, clinical experience remains extremely limited; graft survival after autologous transplantation of adult testicular tissue has been reported, but without evidence of spermatogenic restoration (60). Transplantation of tissue cryopreserved in prepuberty has recently been described in a first-in-human preprint report, with autologous graft survival and histological evidence of spermatogenesis, although restoration of fertility has not been demonstrated (35).

The principal limitation of this approach is the risk of reintroducing malignant cells, particularly in patients with haematological cancers. Because whole tissue fragments are transplanted, any residual malignant cells present in the biopsy may be reimplanted together with the germ cell compartment, representing a major safety concern. Together with unresolved questions regarding graft function, duration of spermatogenic activity, and standardization of the procedure, this concern remains a major barrier to clinical translation (3, 61).

#### *In vitro* spermatogenesis

*In vitro* spermatogenesis aims to generate mature spermatozoa entirely outside the body, using cryopreserved testicular tissue or isolated germ cells as a starting material. Experimental systems include organotypic culture of tissue fragments, three-dimensional culture platforms, and testicular organoid models designed to mimic the seminiferous tubule environment (61, 62, 63, 64, 65).

In humans, partial progression of germ cells through early meiotic stages has been achieved *in vitro*, and xenografting studies have demonstrated advancement to pachytene spermatocytes in grafted infant testicular tissue (64). However, complete spermatogenesis resulting in functional human spermatozoa has not yet been achieved outside the native testicular environment.

A major theoretical advantage of *in vitro* spermatogenesis is oncological safety, as it avoids reimplantation of potentially contaminated tissue. Nevertheless, substantial technical barriers remain, including replication of the complex somatic–germ cell interactions required for meiosis, provision of appropriate endocrine and paracrine signals, and assurance of genetic and epigenetic integrity of any gametes produced. For these reasons, *in vitro* spermatogenesis remains a long-term research objective rather than a realistic near-term clinical option (7).

### Ethical and counselling considerations

Fertility preservation in prepubertal boys raises specific ethical and counselling challenges, primarily related to the experimental nature of TTC and the absence of clinically validated fertility restoration strategies (3). Informed consent is central to ethical practice. Parents or legal guardians must be clearly informed that TTC does not guarantee future fertility and that cryopreserved tissue may ultimately never be used (3). Families should also be informed that TTC is proposed at a stage when future reproductive function cannot yet be clinically evaluated, which makes counselling inherently dependent on estimated risk rather than on direct evidence of gonadal impairment. Counselling should explicitly address the experimental status of all potential applications and the current lack of reported human pregnancies from tissue cryopreserved before puberty. When developmentally appropriate, assent from the child should be sought as part of an age-adapted discussion, acknowledging emerging autonomy without overburdening the child with long-term implications.

Oncological safety represents a major ethical concern, particularly in boys with haematological malignancies. Because current methods cannot completely exclude malignant contamination of cryopreserved tissue, families must be informed that future autologous transplantation strategies may carry unresolved safety risks (40). The timing and context of counselling are also critical. TTC is often discussed shortly after diagnosis, when families face significant emotional stress and urgent treatment decisions. Access to multidisciplinary counselling, including psychological support, is therefore essential to support informed and value-consistent decision-making (48). Because these discussions often take place shortly after diagnosis, written information and, when feasible, repeated counselling sessions may help families make more informed decisions under conditions of high emotional stress (3).

Additional ethical considerations include equity of access to TTC, which is limited to specialized centres, and long-term responsibilities related to tissue storage, future use, and re-counselling once the individual reaches adulthood (3). Clear institutional policies and participation in long-term registries are essential to ensure ethical governance and transparency (3, 48).

## Conclusion

TTC is currently the only fertility preservation option that can be offered to selected prepubertal boys at high risk of treatment-induced infertility, but all strategies for future fertility restoration remain experimental. Its biological rationale is strong, yet its clinical value will ultimately depend on whether safe and effective fertility restoration can be validated for clinical use in humans. TTC should not be regarded as a procedure aimed solely at tissue banking for possible future use but as part of a broader clinical pathway extending from pre-treatment assessment to long-term follow-up during the child’s development. As TTC is offered on the basis of estimated gonadotoxic risk before active fertility can be assessed, and gonadal consequences of treatment and tissue biopsy may emerge only over time, structured baseline assessment and long-term follow-up are essential. In this setting, paediatric endocrine follow-up is also important to monitor growth, pubertal development, and gonadal function and to identify treatment-related abnormalities when they become clinically apparent. When age-appropriate, evaluation of fertility-related outcomes should also be incorporated, primarily through semen analysis and, where useful, supportive surrogate markers, such as follicle-stimulating hormone and inhibin B. TTC therefore fits within an integrated follow-up strategy that does not replace existing survivorship pathways but complements them by adding a fertility preservation perspective to the longitudinal assessment of gonadal outcomes. A comprehensive endocrine and reproductive assessment may also help inform future decisions regarding the preserved tissue, including whether experimental tissue-based approaches should be considered or whether fertility preservation can subsequently rely on conventional sperm-based options. Progress in this field will require advances in fertility restoration techniques, together with multicentre registries and collaborative networks that generate reproducible data on safety and long-term outcomes and support more consistent management of fertility preservation issues in prepubertal boys during endocrine late-effects follow-up.

## Declaration of interest

RTM reports support for the present manuscript from a UK Research and Innovation (UKRI) Future Leaders Fellowship (MR/Y011783/1) and received honoraria from Sandoz and Vertex for invited lectures. VF, FB, and ML declare no competing interests.

## Funding

This work was supported by a UK Research and Innovationhttps://doi.org/10.13039/100014013 (UKRI) Future Leaders Fellowship (grant number MR/Y011783/1). For the purpose of open access, the author has applied a Creative Commons Attribution (CC BY) licence to any Author Accepted Manuscript version arising from this submission.

## Author contribution statement

VF contributed to conceptualization and wrote, reviewed, and edited the manuscript. FB and ML contributed to conceptualization and reviewed and edited the manuscript. RTM contributed to conceptualization, supervision, and review and editing of the manuscript. All authors reviewed and approved the final version of the manuscript and agree to be accountable for all aspects of the work.
